# *Wisteria floribunda* Agglutinin-Positive Mac-2 Binding Protein as a Screening Tool for Significant Liver Fibrosis in Health Checkup

**DOI:** 10.3390/ijms22010040

**Published:** 2020-12-22

**Authors:** Nobuharu Tamaki, Masayuki Kurosaki, Yuka Takahashi, Yoshie Itakura, Sakura Kirino, Kento Inada, Koji Yamashita, Shuhei Sekiguchi, Yuka Hayakawa, Leona Osawa, Mayu Higuchi, Kenta Takaura, Chiaki Maeyashiki, Shun Kaneko, Yutaka Yasui, Kaoru Tsuchiya, Hiroyuki Nakanishi, Jun Itakura, Rohit Loomba, Namiki Izumi

**Affiliations:** 1 Department of Gastroenterology and Hepatology, Musashino Red Cross Hospital, Tokyo 180-8610, Japan; notamaki@health.ucsd.edu (N.T.); kurosaki@musashino.jrc.or.jp (M.K.); sa.kirino@musashino.jrc.or.jp (S.K.); k.inada@musashino.jrc.or.jp (K.I.); ykoji1007@yahoo.co.jp (K.Y.); s.sekikuchi@musashino.jrc.or.jp (S.S.); y.hayakawa@musashino.jrc.or.jp (Y.H.); r.oosawa@musashino.jrc.or.jp (L.O.); mayu621201@yahoo.co.jp (M.H.); tuf029@gmail.com (K.T.); c.maeyashiki@musashino.jrc.or.jp (C.M.); s.kaneko@musashino.jrc.or.jp (S.K.); yutakay@musashino.jrc.or.jp (Y.Y.); tsuchiya@musashino.jrc.or.jp (K.T.); nakanisi@musashino.jrc.or.jp (H.N.); jitakura@musashino.jrc.or.jp (J.I.); 2 Department of Medicine, Division of Gastroenterology and Hepatology, NAFLD Research Center, University of California San Diego, La Jolla, CA 92093, USA; roloomba@health.ucsd.edu; 3 Medical Examination Center, Musashino Red Cross Hospital, Tokyo 180-8610, Japan; y.takahashi@musashino.jrc.or.jp (Y.T.); y.itakura@musashino.jrc.or.jp (Y.I.)

**Keywords:** WFA^+^-M2BP, FIB-4, liver fibrosis, screening

## Abstract

Chronic liver disease is generally widespread, and a test for screening fibrotic subjects in a large population is needed. The ability of *Wisteria floribunda* agglutinin-positive mac-2 binding protein (WFA^+^-M2BP) to detect significant fibrosis was investigated in health checkup subjects in this research. Of 2021 health checkup subjects enrolled in this prospective cross-sectional study, those with WFA^+^-M2BP ≥ 1.0 were defined as high risk. Liver fibrosis was evaluated using magnetic resonance elastography (MRE) in subjects with high risk. The primary outcome was the positive predictive value (PPV) of WFA^+^-M2BP for significant fibrosis (liver stiffness ≥ 2.97 kPa by MRE). This trial was registered with the UMIN clinical trial registry, UMIN000036175. WFA^+^-M2BP ≥ 1.0 was observed in 5.3% of the 2021 subjects. The PPV for significant fibrosis with the threshold of WFA^+^-M2BP at ≥1.0, ≥1.1, ≥1.2, ≥1.3, ≥1.4, and ≥1.5 was 29.2%, 36.4%, 43.5%, 42.9%, 62.5%, and 71.4%, respectively. A WFA^+^-M2BP of 1.2 was selected as the optimal threshold for significant fibrosis among high-risk subjects, and the PPV, negative predictive value, sensitivity, and specificity for significant fibrosis were 43.5%, 84.0%, 71.4%, and 61.8%, respectively. WFA^+^-M2BP ≥ 1.2 was significantly associated with significant fibrosis, with an odds ratio (OR) of 4.04 (95% confidence interval (CI): 1.1–16, *p* = 0.04), but not FIB-4 ≥ 2.67 (OR: 2.40, 95%CI: 0.7–8.6, *p*-value = 0.2). In conclusion, WFA^+^-M2BP is associated with significant fibrosis and could narrow down potential subjects with liver fibrosis. The strategy of narrowing down fibrosis subjects using WFA^+^-M2BP may be used to screen for fibrotic subjects in a large population.

## 1. Introduction

Identification of subjects with liver fibrosis in chronic liver disease (CLD) is important because liver fibrosis strongly correlates with prognosis and mortality [1,2]. However, these subjects have no subjective symptoms, and early detection is difficult. Nonalcoholic fatty liver disease (NAFLD), a cause of CLD, is present in approximately 30% of the population, and CLD is generally widespread in the United States and Europe, as well as in Asia [3,4]. As subjects with liver fibrosis must be identified from a large population, widely available modalities are needed to identify subjects with fibrosis. Serum biomarkers are widely used to evaluate liver fibrosis and prognosis in patients with CLD. As serum markers have high accessibility and low cost, a two-step screening strategy to detect fibrotic subjects in large populations has been suggested [5,6]. In a first-line screening, serum biomarkers would be used to narrow down subjects with potential fibrosis, and imaging modalities or liver biopsy would then be used as a second-line, more detailed test. Although the utility of serum markers for detecting or excluding subjects with fibrosis has been reported [5,6], most studies have been performed in hospital cohorts, and the utility of serum markers in a population-based cohort is limited, while a suitable serum marker for screening a population-based cohort has not yet been determined.

*Wisteria floribunda* agglutinin-positive mac-2 binding protein (WFA^+^-M2BP) is a novel serum fibrosis marker, and diagnostic accuracy for liver fibrosis in chronic hepatitis C, chronic hepatitis B, and NAFLD has been reported [7,8,9,10,11,12]. The pathophysiological role of WFA^+^-M2BP has not been completely elucidated. Hepatic stellate cells (HSCs) are the source of WFA^+^-M2BP, and the WFA^+^-M2BP secreted from HSCs induces Mac-2 expression in Kupffer cells [13]. Furthermore, the co-existence of HSCs and Kupffer cells activates HSCs and increases alpha-smooth muscle actin expression. These findings indicate that WFA^+^-M2BP plays an important role in the progression of liver fibrosis, and WFA^+^-M2BP is associated with the fibrosis stage. Moreover, WFA^+^-M2BP is associated with not only fibrosis, but also hepatocellular carcinoma (HCC) development, the occurrence of liver-related complications, and prognosis [14,15,16,17,18,19]. Therefore, WFA^+^-M2BP may be used as a first-line screening method in a population-based cohort.

FIB-4 is a simple formula for detecting liver fibrosis using age, aspartate aminotransferase (AST), alanine aminotransferase (ALT), and platelet counts, and several studies have reported its high diagnostic accuracy for liver fibrosis, as well as HCC development or occurrence of complications [20,21,22,23,24,25]. Screening for fibrotic NAFLD patients with FIB-4 is currently recommended in the American Association for the Study of Liver Disease guidelines. However, one problem of FIB-4 is that the diagnostic accuracy of FIB-4 decreases in young and elderly subjects, because FIB-4 is affected by age and, therefore, the threshold must be changed according to age [26,27]. On the other hand, WFA^+^-M2BP values are not affected by age [28], and this feature of WFA^+^-M2BP is one advantage when used as a screening method in a large population. However, to date, there has been no report investigating the diagnostic accuracy of WFA^+^-M2BP for fibrosis subjects in a population-based cohort.

To address these knowledge gaps, we conducted a prospective study to identify subjects with liver fibrosis by measuring WFA^+^-M2BP during health checkup screenings, and the diagnostic accuracy of WFA^+^-M2BP was investigated. Furthermore, the diagnostic accuracy of WFA^+^-M2BP and FIB-4 was compared.

## 2. Materials and Methods

### 2.1. Study Protocol

**Figure 1:**
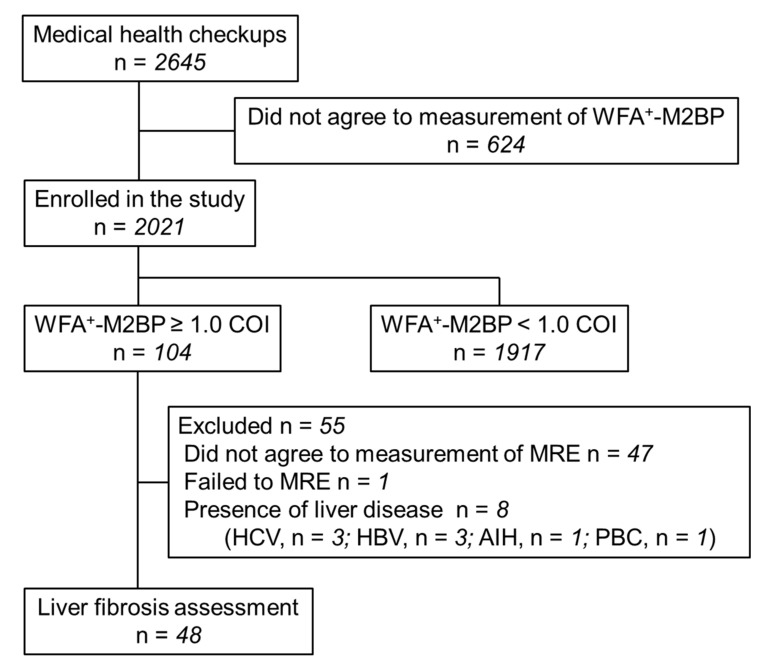
The study flow chart.

This prospective cross-sectional study was registered with the University Hospital Medical Information Network (UMIN) clinical trial registry (UMIN000036175). Health checkup subjects presenting to Musashino Red Cross Hospital between April 2019 and February 2020 were enrolled in the study, and their WFA^+^-M2BP level was measured. Previous studies have reported that the threshold of WFA^+^-M2BP for detecting fibrosis stage 2 or 3 in nonviral hepatitis is 0.7–1.17 and 1.2–1.57, respectively [8]. Based on the results, we set WFA^+^-M2BP ≥ 1.0 as the threshold of a first-line screening. Subjects with WFA^+^-M2BP ≥ 1.0 were defined as high risk, and a further detailed test for liver fibrosis was used. Magnetic resonance elastography (MRE) was performed as the more detailed test to diagnose liver fibrosis in the high-risk subjects (WFA^+^-M2BP ≥1.0). Of 2645 people who underwent a health checkup, 2021 consented to WFA^+^-M2BP measurement and were included in the study (Figure 1). Subjects with already known CLD (chronic hepatitis C, chronic hepatitis B, primary biliary cholangitis (PBC), autoimmune hepatitis, and HCC) were excluded from the analysis. Mortality risk increases in patients with fibrosis stage ≥ 2 (significant fibrosis) in NAFLD patients [1], and clinical trials have been conducted in patients with significant fibrosis. Therefore, the endpoint of this study was to identify subjects with significant fibrosis, and the primary outcome of the study was finding the positive predictive value (PPV) of WFA^+^-M2BP for significant fibrosis. Written informed consent was obtained from each patient. The study protocol was approved by the ethics review committees of Musashino Red Cross Hospital (Approval ID: 30053, 31 January 2019), and conformed to the ethical guidelines of the Declaration of Helsinki.

### 2.2. Clinical and Laboratory Data

Patient age, sex, height, weight, abdominal circumference, and drinking amount were recorded. Blood counts and blood biochemistry tests were conducted using standard methods. The threshold for laboratory data was determined by the upper limit of normal. Physical exams and all biochemical testing were conducted on the same day.

WFA^+^-M2BP measurement was automated using the HISCL-2000 instrument (Sysmex Co., Hyogo, Japan) [29]. The measured WFA^+^-M2BP values of conjugated WFA were indexed with the obtained values using the following equation: Cut-off index (COI) = ([M2BPGi]_sample_ − [M2BPGi]_NC_)/([M2BPGi]_PC_ − [M2BPGi]_NC_), where [M2BPGi]_sample_ is the M2BPGi count of the serum sample, and PC and NC are the positive and negative controls, respectively. The positive control was a calibration solution preliminarily standardized to yield a COI value of 1.0.

The FIB-4 index was calculated by the formula: FIB-4 index = (age [years] × AST [IU/L])/(platelet count [10^9^/L] × (ALT [IU/L])^1/2^) [21], and the threshold of 2.67 was used according to a previous study [30,31]. Similarly, the NAFLD fibrosis score and AST-to-platelet ratio index (APRI) were calculated based on previous studies, and the thresholds of 0.675 and 1.5 were used for the sensitivity analysis [32,33].

### 2.3. Definition of Comorbidity Status

Diabetes mellitus was defined by a fasting plasma glucose level of ≥126 mg/dL, hemoglobin A1c level of ≥6.5%, casual plasma glucose of ≥200 mg/dL, or use of any antihyperglycemic medication. Dyslipidemia and hypertension were diagnosed based on the criteria of metabolic syndrome or the use of medication. Significant alcohol consumption was defined as 30 and 20 g/day in men and women, respectively, according to the guidelines of the Japanese Society of Gastroenterology.

### 2.4. Ultrasound Diagnostics

Conventional ultrasound was performed in all subjects. Ultrasonographic findings of parenchymal brightness, liver-to-kidney contrast, deep beam attenuation, bright vessel walls, and gallbladder wall definition were defined as indicating a fatty liver [34].

### 2.5. Measurement of Liver Stiffness by MRE

MRE was performed using a 1.5 T magnetic resonance system with a superconducting magnet (SignaExcite HD MR 1.5 T; GE Medical Systems, Milwaukee, WI, USA), according to a previously reported method [35,36,37]. All processing steps were automatic, without manual intervention, and yielded quantitative images of tissue shear stiffness in kPa. When measuring liver stiffness, structures such as large blood vessels and the gallbladder were avoided on the constructed liver stiffness map, and a region of interest as large as possible was set to the measurable part. Liver stiffness was measured in three slices, and the average value was used for this study.

### 2.6. Assessment of Liver Fibrosis

Subjects with liver stiffness ≥ 2.97 kPa [38] by MRE were defined as showing significant liver stiffness. One patient who has gastroesophageal varices by esophagogastroduodenoscopy was also defined as showing significant liver stiffness.

### 2.7. Statistical Analysis

Subject characteristics were compared using Fisher’s exact or Student’s *t*-test. A receiver operating characteristic curve (ROC) analysis and Youden index were used to determine the optimal threshold of WFA^+^-M2BP for significant fibrosis. Logistic regression analysis was used for the evaluation of factors associated with significant fibrosis. Statistical significance was defined as *p* < 0.05. Statistical analyses were performed using EZR (Saitama Medical Center, Jichi Medical University, Saitama, Japan) [39] and a graphical user interface for R (The R Foundation for Statistical Computing, Vienna, Austria).

## 3. Results

### 3.1. The Characteristics of the Study Subjects

The study flow chart is shown in Figure 1. WFA^+^-M2BP was measured in 2021 health checkup subjects (average age 57.7 ± 12 years; 1112 men, 909 women; Table 1). The average body mass index (BMI) was 22.6 ± 3.3 kg/m^2^. Fatty liver was noted in 39.4% of the subjects, diabetes mellitus in 7.2%, hypertension in 21.4%, and dyslipidemia in 17.8%. When compared to subjects with WFA^+^-M2BP ≥ 1.0 and those with WFA^+^-M2BP < 1.0, albumin levels and platelet counts were significantly lower in subjects with WFA^+^-M2BP ≥ 1.0, and the presence of diabetes mellitus was significantly higher in subjects with WFA^+^-M2BP ≥ 1.0.

### 3.2. Distribution of WFA^+^-M2BP

**Figure 2:**
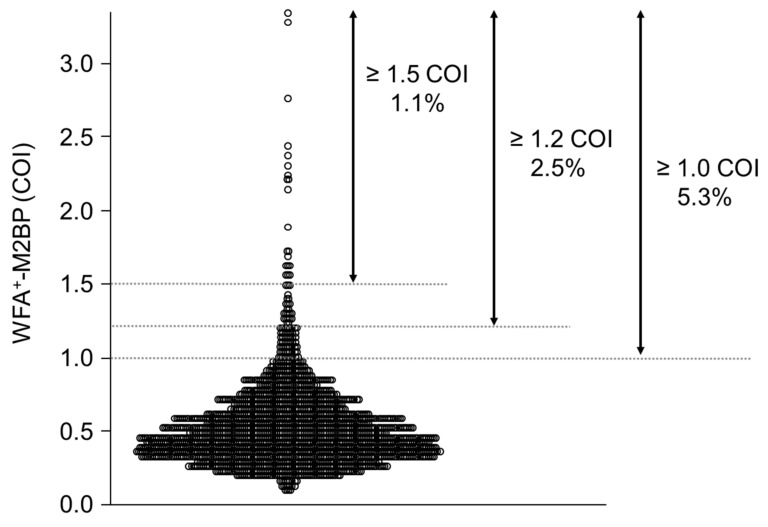
Distribution of WFA^+^-M2BP.

The distribution of WFA^+^-M2BP is shown in Figure 2. The average WFA^+^-M2BP level was 0.53 ± 0.28 COI, and 104 (5.3%) subjects had WFA^+^-M2BP ≥ 1.0. When the threshold of WFA^+^-M2BP was raised to ≥1.1, ≥1.2, ≥1.3, ≥1.4, and ≥1.5 COI, the proportion of subjects with each threshold of WFA^+^-M2BP was 3.6%, 2.5%, 1.6%, 1.3%, and 1.1%, respectively.

### 3.3. Diagnostic Accuracy of WFA^+^-M2BP for Significant Liver Fibrosis

**Figure 3:**
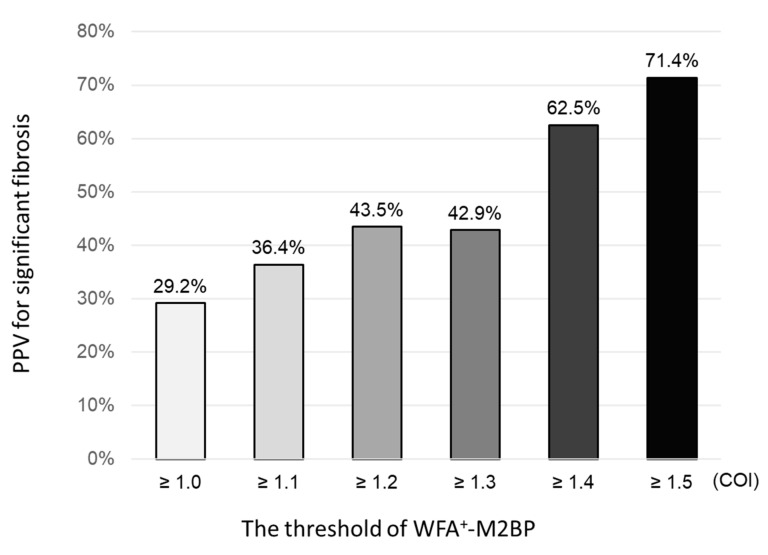
Proportion of significant fibrosis in subjects classified by WFA^+^-M2BP.

We performed MRE in subjects categorized as high risk (WFA^+^-M2BP ≥ 1.0), and 14 subjects were newly diagnosed as having significant liver fibrosis. The PPV for significant fibrosis with WFA^+^-M2BP ≥ 1.0, ≥ 1.1, ≥ 1.2, ≥ 1.3, ≥ 1.4, and ≥ 1.5 COI was 29.2% (14/48), 36.4% (12/33), 43.5% (10/23), 42.9% (6/14), 62.5% (5/8), and 71.4% (5/7), respectively (Figure 3). The PPV of WFA^+^-M2BP for significant fibrosis increased with the threshold of WFA^+^-M2BP.

### 3.4. Characteristics of Newly Diagnosed Subjects with Significant Fibrosis

Of 14 subjects with newly diagnosed significant fibrosis, five (35.7%) had fatty liver, six (42.9%) hypertension, three (21.4%) diabetes mellitus, four (28.6%) dyslipidemia, and four (28.6%) significant alcohol consumption. Regarding background liver disease of the subjects, four had significant alcohol consumption, two had NAFLD, two showed metabolic syndrome without fatty liver, two were positive for anti-mitochondrial antibodies indicating PBC, and four had cryptogenic disease.

### 3.5. Comparison the Diagnostic Accuracy of Serum Fibrosis Markers

**Figure 4:**
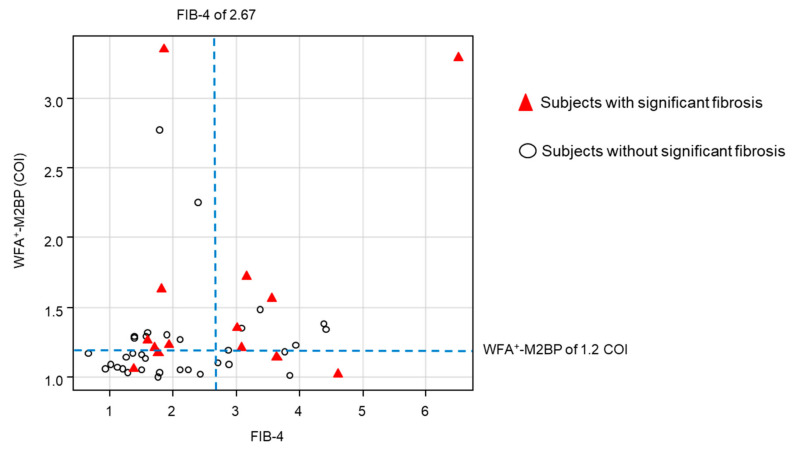
Association between WFA^+^-M2BP, FIB-4, and subjects with significant fibrosis. The blue dotted lines show the threshold of WFA^+^-M2BP and FIB-4. The red triangles show the subjects with significant fibrosis. The white circles show the subjects without significant fibrosis.

A WFA^+^-M2BP of 1.2 COI was selected as the optimal threshold for significant fibrosis by ROC analysis and the Youden index. The association between WFA^+^-M2BP, FIB-4, and subjects with significant fibrosis is shown in Figure 4. The PPV, negative predictive value (NPV), sensitivity, and specificity of WFA^+^-M2BP for significant fibrosis were 43.5% (10/23), 84.0% (21/25), 71.4% (10/14), and 61.8% (21/34), respectively (Table 2). When examined, the diagnostic accuracy of FIB-4 ≥ 2.67, PPV, NPV, sensitivity, and specificity were 41.2% (7/17), 77.4% (24/31), 50.0% (7/14), and 70.6% (24/34), respectively. Similarly, the PPV, NPV, sensitivity, and specificity of the NAFLD fibrosis scores (≥0.675) were 60.0% (3/5), 74.4% (32/43), 21.4% (3/14), and 94.1% (32/34), respectively. No subject met the criteria of APRI ≥ 1.5. The AUROC of WFA^+^-M2BP, FIB-4, and NAFLD fibrosis score for significant fibrosis were 0.681, 0.665, and 0.599, respectively, and the AUROC was higher in WFA^+^-M2BP than in the FIB-4 and NAFLD fibrosis scores.

### 3.6. Factors Associated with Significant Fibrosis

Factors associated with significant fibrosis were examined in subjects who received MRE assessment. WFA^+^-M2BP ≥ 1.2 COI was significantly associated with significant fibrosis, with an odds ratio (OR) of 4.04 (95% confidence interval (CI): 1.1–16, *p* = 0.04, Table 3). On the other hand, FIB-4 ≥ 2.67 (OR: 2.40, 95%CI: 0.7–8.6, *p*-value = 0.2) and NAFLD fibrosis score (OR: 4.36, 95%CI: 0.6–29, *p*-value = 0.1) were not associated with significant fibrosis. Age, sex, BMI, other laboratory data, and presence of comorbidities were also not associated with significant fibrosis.

## 4. Discussion

### 4.1. Main Findings

In this prospective study, we identified undiagnosed subjects of significant liver fibrosis using WFA^+^-M2BP screening. When using the threshold of WFA^+^-M2BP ≥ 1.2 COI, the population was narrowed down to 2.5%, and among the subjects with WFA^+^-M2BP ≥ 1.2 COI, about half of the subjects had significant fibrosis. Furthermore, WFA^+^-M2BP ≥ 1.2 COI demonstrated a four-times-higher odds of significant fibrosis even among the high-risk subjects (WFA^+^-M2BP ≥ 1.0 COI). On the other hand, FIB-4 was not associated with significant fibrosis, nor were other laboratory data and the presence of comorbidities; WFA^+^-M2BP was the only factor associated with significant fibrosis. These results indicated that the strategy of narrowing down fibrosis subjects using WFA^+^-M2BP may be used to screen for subjects with liver fibrosis in a large population.

### 4.2. Context with Published Literature

CLD, such as NAFLD, occurs widely in the general population [3], and identification of fibrotic subjects is needed. To screen a large population, the use of an easily available blood test has been proposed [5,6]. Therefore, we conducted this prospective study. The FIB-4 index requires only standard serum tests, and is widely used to screen for significant fibrosis. However, FIB-4 was not associated with significant fibrosis in this study. As the FIB-4 index includes age in the formula and is affected by age, an age-dependent threshold has been proposed [26,27]. This limitation of FIB-4 might affect its diagnostic accuracy. On the other hand, one advantage of WFA^+^-M2BP is that it is not affected by age [28], and WFA^+^-M2BP was the only factor associated with significant fibrosis in this study. Previous studies have reported that the threshold of WFA^+^-M2BP for detecting fibrosis stage 2 or 3 in nonviral chronic hepatitis patients is 0.7–1.17 COI and 1.2–1.57 COI, respectively [8]. A WFA^+^-M2BP of 1.2 COI was selected as the optimal threshold for detecting significant fibrosis in this population-based study, and the threshold is reasonable compared to previous studies. These results demonstrated the validity and significance of using WFA^+^-M2BP as a population-based screening tool.

The strength of this study is that the utility of WFA^+^-M2BP for identifying fibrotic subjects was demonstrated in a population-based cohort. Although several studies have reported the utility of WFA^+^-M2BP for identifying liver fibrosis, these studies were conducted in patients with already known CLD (a hospital cohort). Due to the increase in CLD, it is necessary to verify the ability of serum fibrosis markers to diagnose liver fibrosis in the general population, but such studies have not yet been conducted. To avoid the potential selection bias of a hospital cohort, we targeted subjects who underwent a health checkup as being representative of the general population, and demonstrated the utility of WFA^+^-M2BP for detecting fibrotic subjects in this large cohort.

Ultrasound-based modalities to evaluate pathological findings in the liver have been developing in recent years [40,41,42]. Some studies have investigated population-based screening trials with transient elastography (TE), and identified 5.8 to 7.5% high-risk subjects (liver stiffness by TE > 8 KPa) [43,44]. However, the limitation of these studies was that further detailed examination (liver biopsy or MRE) was not carried out, and the actual percentage of significant fibrosis subjects is therefore not known. One advantage of our study was that MRE was performed as a standard method for examining fibrosis in subjects with high risk. When we used the threshold of WFA^+^-M2BP ≥ 1.2 COI, 2.5% of the study subjects required further testing, and previously undiagnosed subjects with significant liver fibrosis were identified. As WFA^+^-M2BP can be measured easily by a simple blood test and no specific device is required, screening of high-risk subjects with WFA^+^-M2BP is useful in clinical practice. However, further investigations comparing the diagnostic accuracy and cost-effectiveness between TE and WFA^+^-M2BP as a screening tool for liver fibrosis are necessary.

### 4.3. Strengths and Limitations

The strengths of the study are that large numbers of subjects (more than 2000 subjects) were enrolled prospectively in the study, and 14 subjects with significant fibrosis were newly diagnosed. However, some limitations exist in this study. As liver fibrosis was not evaluated in the subjects with WFA^+^-M2BP < 1.0, the false-negative rate of significant fibrosis in subjects with WFA^+^-M2BP < 1.0 was not clear. Previous reports have demonstrated that the NPV of WFA^+^-M2BP for advanced fibrosis and cirrhosis in NAFLD patients was 88.3–91.9% and 93.7–96.4%, respectively [10,11,28,45]. Moreover, based on the high NPV of serum fibrosis markers, further investigation is not recommended for subjects with low serum fibrosis markers in the American Association for the Study of Liver Disease and the European Association for the Study of the Liver guidelines [46,47]; these subjects will be followed up after a few years. We conducted this study based on these recommendations. However, there are no data on the distribution of significant fibrosis in a population-based cohort, and investigating the distribution is needed in future studies, including through FIB-4 or ultrasound-based elastography. Moreover, MRE was used as the standard method for the evaluation of fibrosis. Although liver biopsy is a gold standard for the diagnosis of liver fibrosis, it was not used in this study. However, there is an inter- and intra-observer discrepancy in the evaluation of fibrosis by liver biopsy [48,49], and MRE has been used as the method for evaluation of endpoints in many clinical trials [36,50]. These data provide justification for using MRE to assess liver fibrosis in this study. However, further investigations that include liver biopsy are needed.

### 4.4. Future Implications

We demonstrated that WFA^+^-M2BP may be helpful as a population-based screening tool of liver fibrosis. The distribution and background of liver fibrosis differ by race and region. It is also known that WFA^+^-M2BP values differ depending on the background of liver disease, and the threshold of WFA^+^-M2BP for detecting significant fibrosis is higher in patients with viral hepatitis than in those with nonviral hepatitis [8]. Therefore, verification of the diagnostic ability of WFA^+^-M2BP in other population-based cohorts, like those with a high prevalence of viral hepatitis, is necessary, and may strengthen the evidence for the use of WFA^+^-M2BP as a population-based screening tool of liver fibrosis. One limitation of the study is the lack of liver fibrosis assessment in subjects of WFA^+^-M2BP < 1.0 COI. An investigation of the prevalence of significant fibrosis in subjects with low serum fibrosis markers would provide clinically and epidemiologically essential information for further understanding CLD, and further studies in this regard are important. Furthermore, the utility of other liver fibrosis markers, such as the N-terminal propeptide of type 3 procollagen or autotaxin [51,52], has been reported recently, and it will be necessary to compare the diagnostic ability and cost-effectiveness between these fibrosis markers, ultrasound-based elastography, and WFA^+^-M2BP in the general population in the future.

CLD is the risk of hepatocellular carcinoma (HCC) development, cardiovascular disease, and mortality. We previously reported that a high level of WFA^+^-M2BP is associated with progression to decompensated cirrhosis and mortality [16]. Furthermore, a time course increase in WFA^+^-M2BP is associated with HCC development [14]. Early detection of HCC development is an important clinical issue, but no HCC patients were included in this study. Therefore, further follow-up investigation is needed to evaluate the association between WFA^+^-M2BP and its changes and HCC development or prognosis.

## 5. Conclusions

In conclusion, WFA^+^-M2BP is associated with significant fibrosis and could narrow down potential subjects with liver fibrosis. Our strategy of narrowing down fibrosis subjects using WFA^+^-M2BP could be used to screen for subjects with liver fibrosis in a large population.

## Figures and Tables

**Table 1:** Subject characteristics.

	All Subjects	Subjects with WFA^+^-M2BP < 1.0 COI	Subjects with WFA^+^-M2BP ≥ 1.0 COI	*p* Value
	(*n* = 2021)	(*n* = 1917)	(*n* = 104)	
Age, years	57.7 ± 12	57.1 ± 12	67.1 ± 13	<0.01
Sex, male/female	1112/909	1059/858	53/51	0.4
BMI, kg/m^2^	22.6 ± 3.3	22.6 ± 3.3	23.1 ± 3.2	0.1
Abdominal circumference, cm	84.5 ± 9.7	84.4 ± 9.8	86.6 ± 8.5	0.02
Albumin, g/dL	4.3 ± 0.26	4.3 ± 0.26	4.1 ± 0.26	<0.01
AST, IU/L	22.8 ± 7.8	22.6 ± 7.6	26.7 ± 8.9	<0.01
ALT, IU/L	21.2 ± 12	21.1 ± 12	23.0 ± 13	0.2
Gamma-glutamyl transpeptidase, IU/L	33.7 ± 33	33.3 ± 32	41.6 ± 55	0.01
Total cholesterol, mg/dL	207 ± 32	207 ± 32	200 ± 34	0.03
Triglycerides, mg/dL	101 ± 67	100 ± 68	105 ± 61	0.5
Platelet counts, × 10^9^/L	231 ± 52	233 ± 51	211 ± 58	<0.01
WFA^+^-M2BP, COI	0.53 ± 0.28	0.48 ± 0.18	1.34 ± 0.45	<0.01
FIB-4	1.38 ± 0.7	1.34 ± 0.6	2.04 ± 1.0	<0.01
NAFLD fibrosis score	−1.977 ± 1.2	−2.027 ± 1.1	−1.068 ± 1.3	<0.01
APRI	0.27 ± 0.12	0.27 ± 0.11	0.37 ± 0.18	<0.01
Hemoglobin A1c, %	5.79 ± 0.48	5.78 ± 0.46	5.94 ± 0.67	<0.01
Fatty liver, *n* (%)	797 (39.4%)	749 (39.1%)	48 (46.2%)	0.2
Hypertension, *n* (%)	432 (21.4%)	393 (20.5%)	39 (37.5%)	<0.01
Diabetes mellitus, *n* (%)	146 (7.2%)	129 (6.7%)	17 (16.3%)	<0.01
Dyslipidemia, *n* (%)	359 (17.8%)	334 (17.4%)	25 (24.0%)	0.08
Significant alcohol consumption, *n* (%)	418 (20.8%)	400 (20.9%)	18 (17.3%)	0.5

BMI—body mass index; AST—aspartate aminotransferase; ALT—alanine aminotransferase; WFA^+^-M2BP—Wisteria floribunda agglutinin-positive mac-2 binding protein; COI—cut-off index; NAFLD—nonalcoholic fatty liver disease; APRI—aspartate aminotransferase-to-platelet ratio index. Data are shown as the mean ± standard deviation.

**Table 2:** The diagnostic accuracy of serum fibrosis markers for significant fibrosis.

	PPV	NPV	Sensitivity	Specificity
WFA^+^-M2BP ≥ 1.2 COI	43.5%	84.0%	71.4%	61.8%
FIB-4 ≥ 2.67	41.2%	77.4%	50.0%	70.6%
NAFLD fibrosis score ≥ 0.675	60.0%	74.4%	21.4%	94.1%
APRI ≥ 1.5	No subject met the criteria of APRI ≥ 1.5			

PPV—positive predictive value; NPV—negative predictive value; WFA+-M2BP—Wisteria floribunda agglutinin-positive mac-2 binding protein; COI—cut-off index; NAFLD—nonalcoholic fatty liver disease; APRI—aspartate aminotransferase-to-platelet ratio index.

**Table 3:** Factors associated with significant fibrosis.

	OR	95% CI	*p*-Value
Age (per 10 years)	0.75	0.4–1.3	0.3
Sex (female)	0.71	0.2–2.6	0.6
BMI, kg/m^2^	1.16	0.9–1.5	0.2
Albumin < 4.1 g/dL *	2.44	0.7–9.2	0.2
AST > 38 IU/L *	4.4e7	0.0–inf	0.9
ALT > 43 IU/L *	2.54	0.2–43	0.5
Gamma-glutamyl transpeptidase >80 IU/L for male and >40 IU/L for female *	2.59	0.6–10	0.2
Platelet counts < 160 (10^9^/L) *	1.54	0.4–5.9	0.5
Presence of fatty liver	0.79	0.2–2.9	0.7
Presence of hypertension	1.07	0.3–3.8	0.9
Presence of diabetes mellitus	2.82	0.5–16	0.2
Presence of dyslipidemia	1.30	0.3–5.3	0.7
Presence of significant alcohol consumption	4.13	0.8–21	0.1
WFA^+^-M2BP ≥ 1.2 COI	4.04	1.1–16	0.04
FIB-4 ≥ 2.67	2.40	0.7–8.6	0.2
NAFLD fibrosis score ≥ 0.675	4.36	0.6–29	0.1

Bold characters indicate factors with *p* value < 0.05. * The threshold was determined by the upper limit of normal. BMI—body mass index; AST—aspartate aminotransferase; ALT—alanine aminotransferase; WFA+-M2BP—Wisteria floribunda agglutinin-positive mac-2 binding protein; COI—cut-off index; OR—odds ratio; CI—confidence interval; NAFLD—nonalcoholic fatty liver disease. Data are shown as the mean ± standard deviation.

## Abbreviations

CLD	chronic liver disease
NAFLD	nonalcoholic fatty liver disease
WFA^+^-M2BP	*Wisteria floribunda* agglutinin-positive mac-2 binding protein
HSC	hepatic stellate cell
HCC	hepatocellular carcinoma
AST	aspartate aminotransferase
ALT	alanine aminotransferase
MRE	magnetic resonance elastography
COI	cut-off index
PBC	primary biliary cholangitis
ROC	receiver operating characteristic curve
BMI	body mass index
PPV	positive predictive value
NPV	negative predictive value
OR	odds ratio
CI	confidence interval
TE	transient elastography
HCC	hepatocellular carcinoma
APRI	aspartate aminotransferase-to-platelet ratio index
